# Exosome-derived noncoding RNAs in gastric cancer: functions and clinical applications

**DOI:** 10.1186/s12943-021-01396-6

**Published:** 2021-07-30

**Authors:** Xiao-Huan Tang, Ting Guo, Xiang-Yu Gao, Xiao-Long Wu, Xiao-Fang Xing, Jia-Fu Ji, Zi-Yu Li

**Affiliations:** 1 Key Laboratory of Carcinogenesis and Translational Research (Ministry of Education/Beijing), Peking University Cancer Hospital & Institute, No. 52 Fu-Cheng Road, Hai-Dian District, Beijing, 100142 P.R. China; 2 Department of Gastrointestinal Cancer Center, Ward I, Peking University Cancer Hospital & Institute, No. 52 Fu-Cheng Road, Hai-Dian District, Beijing, 100142 P.R. China

**Keywords:** Exosome, Gastric cancer, ncRNA, Progression, Biomarker

## Abstract

Exosomes are a subpopulation of the tumour microenvironment (TME) that transmit various biological molecules to promote intercellular communication. Exosomes are derived from nearly all types of cells and exist in all body fluids. Noncoding RNAs (ncRNAs) are among the most abundant contents in exosomes, and some ncRNAs with biological functions are specifically packaged into exosomes. Recent studies have revealed that exosome-derived ncRNAs play crucial roles in the tumorigenesis, progression and drug resistance of gastric cancer (GC). In addition, regulating the expression levels of exosomal ncRNAs can promote or suppress GC progression. Moreover, the membrane structures of exosomes protect ncRNAs from degradation by enzymes and other chemical substances, significantly increasing the stability of exosomal ncRNAs. Specific hallmarks within exosomes that can be used for exosome identification, and specific contents can be used to determine their origin. Therefore, exosomal ncRNAs are suitable for use as diagnostic and prognostic biomarkers or therapeutic targets. Regulating the biogenesis of exosomes and the expression levels of exosomal ncRNAs may represent a new way to block or eradicate GC. In this review, we summarized the origins and characteristics of exosomes and analysed the association between exosomal ncRNAs and GC development.

## Background

Gastric malignancy is a commonly diagnosed cancers and a leading cause of cancer-related deaths. In 2020, the number of new cases of gastric cancer (GC) ranked fifth among all cancers and was the fourth most common cause of cancer-related death [1]. Although the combination of surgery, chemotherapy, radiotherapy and immunotherapy has improved survival in these patients, most of them still experience relapse and metastasis, which leads to a poor prognosis. Over the last few years, the proposal of several molecular types of GC has also provided potential conditions for personalized treatment [2]. Studies on the tumour microenvironment (TME) have provided a novel understanding of tumour growth and new pathways to treat cancer [3]. Among all components of the TME, exosomes are an important integral part.

Exosomes, also termed intraluminal vesicles (ILVs), are a subpopulation of extracellular vesicles (EVs) with a 30–150 nm diameter derived from multivesicular bodies (MVBs) that transmit cellular molecular constituents, including proteins, DNA, lipids, messenger RNAs (mRNAs), microRNAs (miRNAs), long noncoding RNAs (lncRNAs) and circular RNAs (circRNAs), to promote intercellular communication [4, 5]. According to data from the ExoCarta database (http://www.exocarta.org), identified exosome contents include 9769 proteins, 3408 mRNAs, 2838 miRNAs and 1116 lipids. In 1986, Johnstone et al. first observed and harvested exosomes during the culture of sheep reticulocytes. However, due to their lack of exosomal structure and biological activity, they were regarded as “garbage” produced along with the shedding of specific membrane functions [6]. In 1996, Raposo and colleagues found that murine and human B lymphocytes secreted exosomes that contained MHC class II and transferred MHC molecules to the plasma membrane, inducing antigen-specific MHC class II-restricted T cell responses. These results revealed that exosomes exert effects on antigen presentation in vivo [7]. In 2007, Valadi and coworkers reported that there were numerous mRNAs and microRNAs in exosomes from human and murine mast cells that can be transferred to other cells [8]. Moreover, these delivered exosomal mRNAs can be translated in recipient cells, suggesting that cell–cell communication can occur through RNA transfer via exosomes. Subsequently, exosomal investigation attracted increasing attention from researchers worldwide [8]. Later, researchers found that cancer cells and other stromal cells in the TME also secreted exosomes and modulated tumour progression through exosome-mediated molecular exchanges [9, 10]. Subsequently, an increasing number of molecules, including DNA, proteins, RNA and peptides, have been found to be transferred among different cells by exosomes [11]. Recently, studies found that exosomes can be secreted by nearly all types of cells and exist in all body fluids, including plasma, serum, lymph, gastric juice, urine, bile, saliva, bronchial fluid, breast milk, cerebral spinal fluid (CSF), amniotic fluid, synovial fluid and semen [12–25]. Over the past few years, an increasing number of functional molecules within exosomes have been identified. In addition, studies have shown that exosomes and their cargos exert important effects on cancer initiation and progression [26].

Among all exosomal cargos, noncoding RNAs (ncRNAs) are one of the most abundant contents. In addition, previous studies have found that some molecules are specifically transferred into exosomes, including proteins and ncRNAs [27]. For example, a previous study reported that heterogeneous nuclear ribonucleoprotein A2B1 (hnRNPA2B1), a ubiquitously expressed RNA-binding protein, regulates the transport and subcellular localization of certain mRNAs in neurons [28]. Villarroya-Beltri and coworkers found that hnRNPA2B1 guides the loading of a specific subset of miRNAs and mRNAs into exosomes [29]. In addition, major vault protein (MVP) mediates the sorting of exosomal miR-193a and promotes colon cancer progression [30]. Furthermore, they observed that there were correspondingly highly represented miRNAs in exosomes and cells, suggesting that specific repertoires of miRNAs are sorted into exosomes. These specific cellular signals may also contain potential prognostic and diagnostic information for cancer. Recently, various exosomal ncRNAs were found to modulate GC initiation, progression and chemoresistance. Furthermore, exosomes play a vital role in the interaction between GC progression and *Helicobacter pylori* (*H. pylori*) infection, the primary risk factor for GC. Therefore, targeting exosomes or these exosome-derived ncRNAs may represent a new way to treat GC. In this review, we summarize the characteristics of exosomes and the detailed association between exosomal ncRNAs and GC, hoping to contribute to the understanding of exosomes and exosomal ncRNAs and suggest novel pathways related to exosomal ncRNAs that can be regulated to block or eradicate GC.

### Exosomes origin

**Fig. 1:**
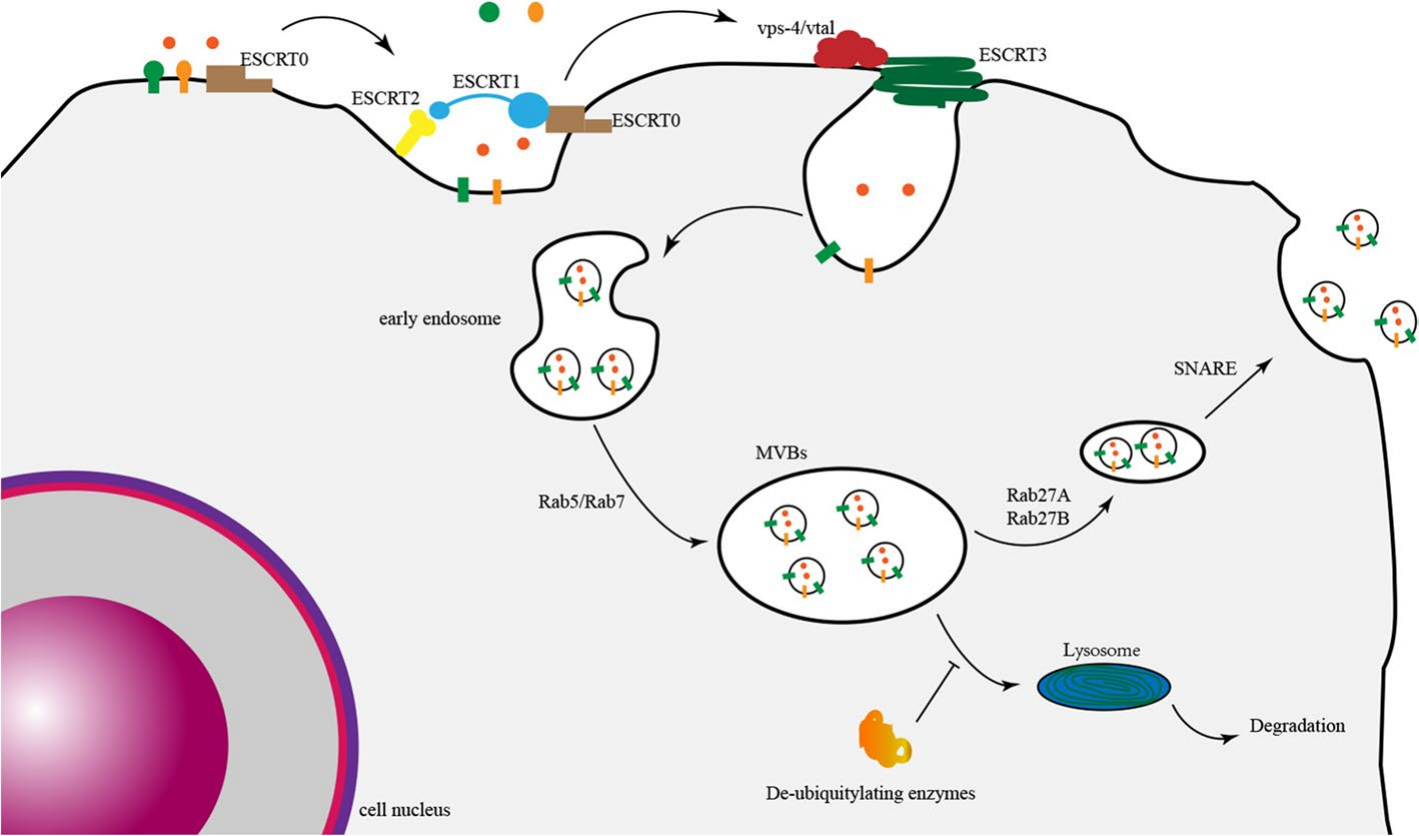
The molecular mechanisms of exosomal biogenesis and metabolism: ESCRT-0 first recognizes ubiquitinated cargo; ESCRT-I and ESCRT-II complexes form an ESCRT-cargo-enriched zone; ESCRT-III recruits deubiquitination machinery and packages cargo into maturing vesicles and promotes vesicle budding. Next, these inward buds form early endosomes and further mature into MVBs. Rap5 and Rap7 promote the development of MVBs from early endosomes. Rab27A and Rab27B induce the translocation of MVBs to the cell periphery, and then the SNARE complex mediates membrane fusion between MVBs and the plasma membrane to release exosomes. A deubiquitinating enzyme suppresses the degradation of MVBs in the lysosome

The exact mechanism of exosome formation is still not well understood, and the endosomal sorting complex required for transport (ESCRT) is a classic pathway. In 2001, the ESCRT-I complex was identified and shown to participate in the formation and sorting of exosomes [31]. Next, additional related complexes, such as ESCRT-II and ESCRT-III, and their functions were identified [32, 33]. The ESCRT pathway can act as a machine for the recognition of ubiquitinated cargo and membrane deformation (Fig. 1). Briefly, ESCRT-0 recognizes ubiquitinated cargo and initiates this pathway; ESCRT-I and ESCRT-II complexes bind to each other and cargo to form an ESCRT-cargo-enriched zone; then, the ESCRT-II complex promotes the assembly of ESCRT-III; finally, ESCRT-III recruits deubiquitination machinery and packages cargo into maturing vesicles and promotes vesicle budding [34]. Finally, these inward buds form early endosomes and further mature into MVBs [35, 36]. The small GTPases Rap5 and Rap7 play a crucial role in the development of MVBs from early endosomes [37]. MVBs can be degraded by lysosomes or fuse with the plasma membrane to release their contents, including exosomes [38–40]. The factors that influence the direction of MVB metabolism are still not clear, but a previous study found that MVBs enriched with cholesterol tended to be secreted into the extracellular space, while others tended to be degraded [41]. In addition, Rab27A and Rab27B, two Rab family components, induce the translocation of MVBs to the cell periphery, and then the sensitive factor attachment protein receptor (SNARE) complex mediates membrane fusion between MVBs and the plasma membrane to release exosomes [42–44]. The endosome-related deubiquitinating enzyme ubiquitin-specific peptidase 8 (USP8) may inhibit the degradation of MVBs by regulating APP intracellular domain (AICD) protein levels [45].

### Hallmarks of exosomes

**Fig. 2:**
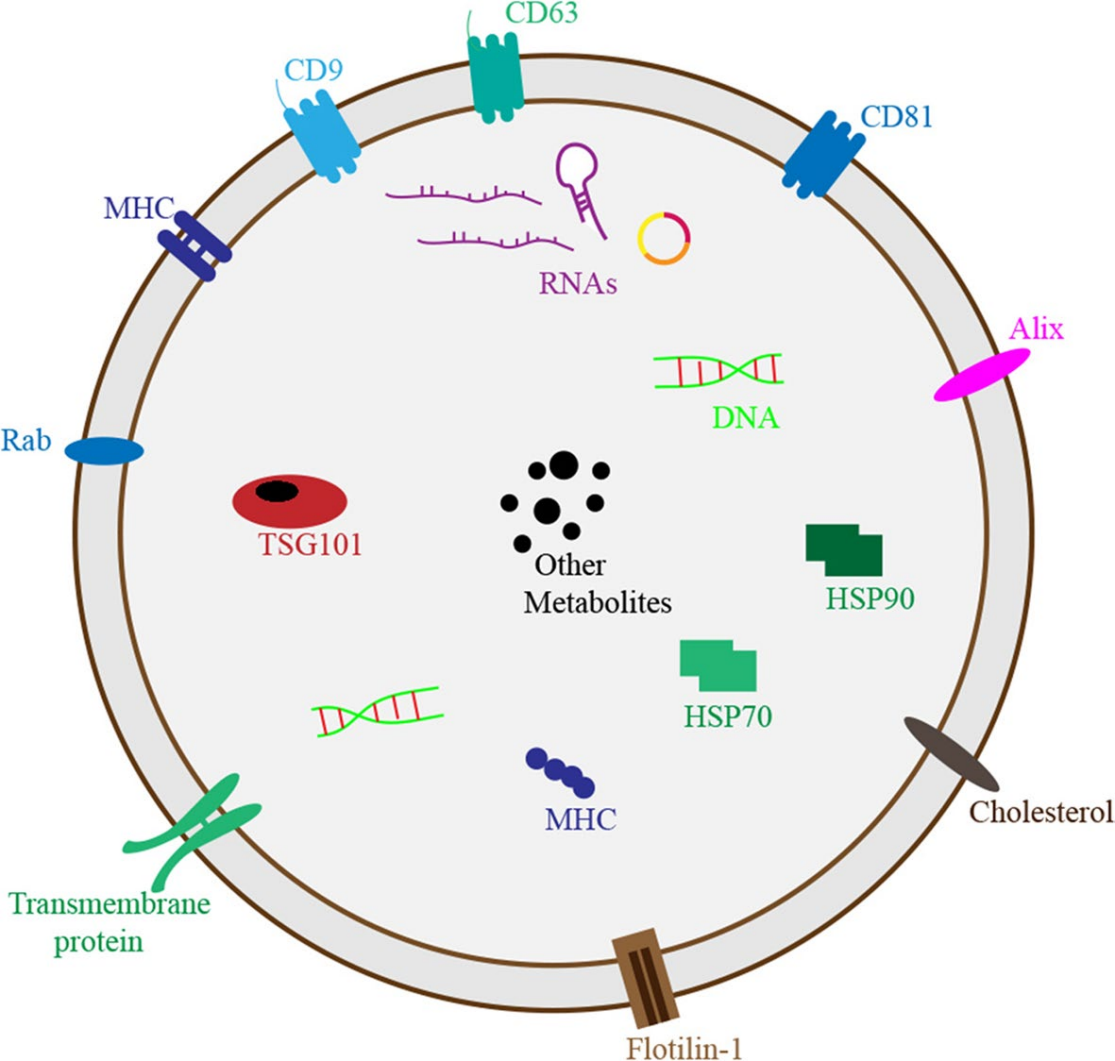
The hallmarks and cargos of exosomes

Analysis of proteins in exosomes revealed that some proteins derived from cells or tissues are specifically packaged into exosomes, while some are common (Fig. 2). In addition to some common molecular compositions, unique cellular function-related subsets of proteins also exist in exosomes [27]. Adhesion molecules, including integrins, tetraspanins, CAMs, transferrin receptors (TfR) on reticulocytes and major histocompatibility complex (MHC) on dendritic cells and B cells, are typical molecules of specific proteins in exosomes [27]. Some proteins enriched in exosomes belong to nonspecific molecules and are usually regarded as markers for the identification of exosomes, such as CD9, CD63, CD81, ALG2-interacting protein X (ALIX), heat shock protein 70 (HSP70) and tumour susceptibility gene 101 protein (TSG101) [27, 46]. Although the identity of specific biomarkers for exosomes remain elusive due to the lack of standard extraction methods, researchers can identify exosomes using the abovementioned hallmarks and trace their origins on the basis of specific proteins.

### Exosomal ncRNAs and GC

In recent decades, the critical roles of ncRNAs in cancer progression, metastasis and drug resistance have been reported by substantial studies [47]. Recently, studies have found that numerous oncogenic molecules, including miRNAs, circular RNAs and lncRNAs, can be transferred into tumour cells by exosomes to promote therapeutic resistance and further induce cancer progression [48, 49]. A growing amount of evidence has revealed that exosomal ncRNAs exert crucial functions in GC tumorigenesis, progression, metastasis, angiogenesis and chemoresistance. In 2009, the functions of exosomes in GC progression were first identified by Qu and coworkers [50]. They found that SGC-7901-secreted exosomes promoted proliferation of SGC-7901 and BGC-823 cells by activating the AKT signalling pathway. Subsequently, several studies further confirmed that exosomes promoted GC progression in an autocrine manner [50]. In 2010, Ohshima et al. first found that the miRNA let-7 derived from GC cells was enriched in the extracellular environment via exosomes and promoted GC metastasis [51]. Subsequently, Wang and colleagues reported that GC tissue-derived mesenchymal stem cells (GC-MSCs) transferred miR-221 via exosomes to HGC-27 cells, promoting the proliferation and migration of HGC-27 cells [52]. In addition, exosomes carrying lncHEIH released by GC cells promote the malignant transformation of normal gastric cells [53]. Thereafter, the interaction between exosomal ncRNAs and GC attracted increasing attention among researchers.

### Exosomal ncRNAs and *Helicobacter pylori* (*H. pylori*)

Infection with *H. pylori* is the most dominant risk factor for GC [54], and almost all patients with noncardia GC have this bacterium [55, 56]. However, the precise mechanisms between *H. pylori* infection and the tumorigenesis of GC are still not well defined. Therefore, exploring the interactions between *H. pylori* infection and GC development has substantial implications for preventing GC. Many studies have shown that ncRNAs participate in biological processes. For example, *H. pylori* infection enhances expression of NLRP3, an important inflammasome component, by decreasing miR-22 levels to trigger uncontrolled proliferation of gastric epithelial cells [57]. Tsai and colleagues found that miR-18a-3p and miR-4286 were significantly upregulated in *H. pylori*-associated GC [58]. Furthermore, overexpression of miR-18a-3p and miR-4286 promoted cancer cell proliferation and motility by targeting BZRAP1. Recently, exosomes have gradually been recognized as an important link between *H. pylori* infection and tumorigenesis. Shimoda et al. reported that exosomes can act as nanocarriers to deliver the virulence factor CagA of *H. pylori* into gastric epithelial cells to induce elongated cell shape in these cells [59]. In addition, gastric epithelial cells with enriched CagA also secrete exosomes containing CagA. Another study found that *H. pylori* infection increased the expression of exosomal activated mesenchymal-epithelial transition factor (MET) protein in macrophages to promote macrophage acquisition of a tumorigenesis-promoting phenotype, promoting GC progression. These findings suggest that exosome-mediated molecular communication plays an important role in the crosstalk between *H. pylori* infection and GC. As the most enriched molecules in exosomes, ncRNAs must also play similar roles, although there is currently no related literature on this, representing a promising direction for future research.

### Exosomal ncRNAs and GC progression

Cancer cells detach from the primary cancer nest to enter the circulatory system through vascular and lymphatic vessels, which facilitates tumour metastasis [60]. To avoid blocking the extracellular matrix (ECM) and to develop a tumour-friendly microenvironment, cancer cells release bioactive factors that mediate communication between tumour cells and stromal cells to create favourable conditions for tumour cell invasion and migration [60, 61]. Exosomes play an important role in this process by delivering DNA, lipids and ncRNAs.

**Fig. 3:**
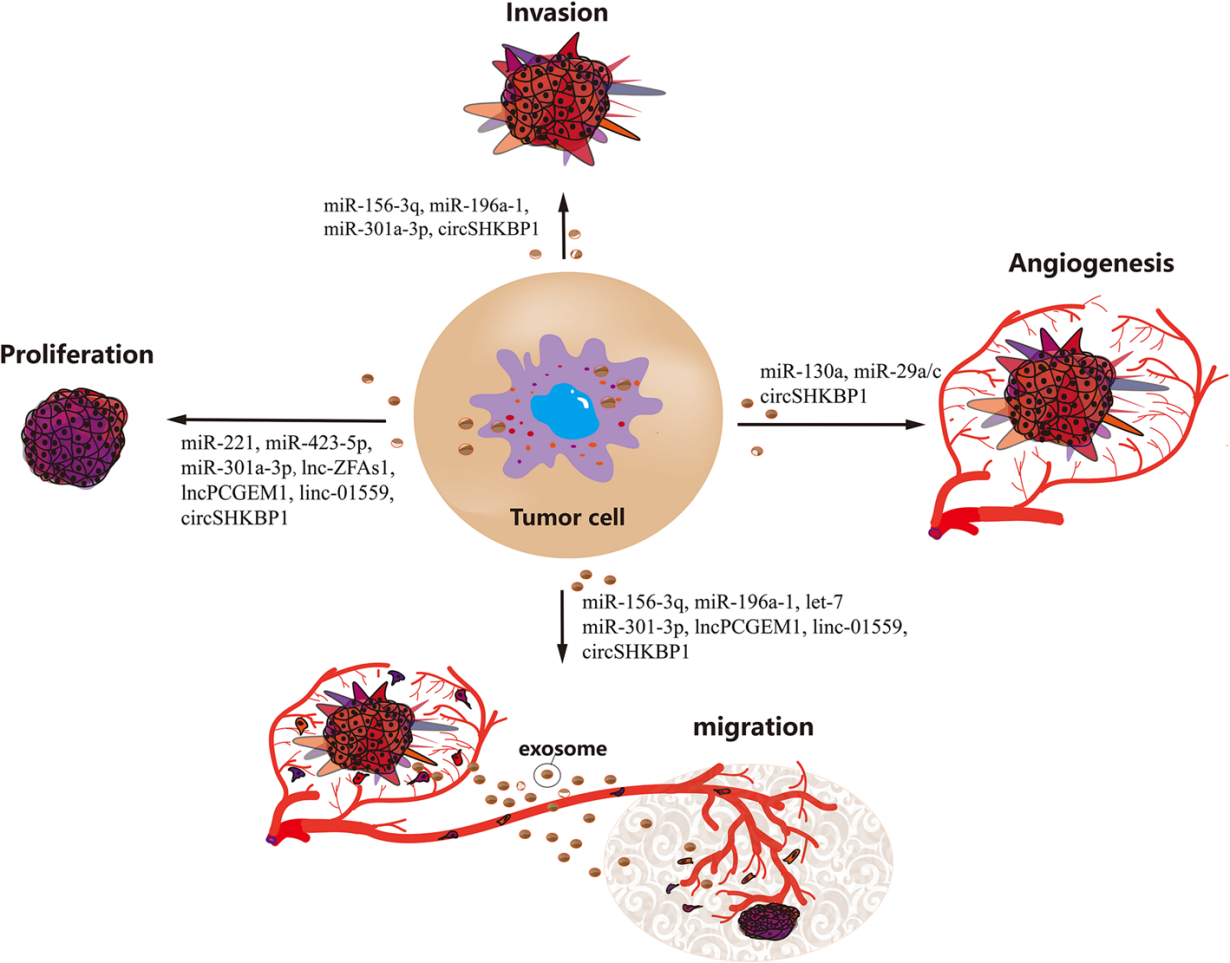
Exosomal ncRNAs regulate the proliferation, invasion, angiogenesis and migration of GC cells

Several ncRNAs contained in exosomes that promote the proliferation, invasion, angiogenesis and migration of GC cells have been identified (Fig. 3). For example, GC-MSCs significantly enhance the proliferative and migration abilities of the GC cell line HGC-27 by delivering exosomal miR-221 in a paracrine manner [52]. Exosomal miR-15b-3p promotes the migration and invasion of GC cells by targeting DYNLT1, caspase-3 and caspase-9 [62]. GC cells with low invasion transfected with exosomal miR-196a-1 exhibited enhanced invasion, and mechanistic research found that exosomal miR-196a-1 exerts modulatory effects by targeting SFRP1 [63]. In addition, angiogenesis is closely related to the invasion and migration of GC cells, and Yang et al. found that SGC exosome-delivered miR-130a enhances angiogenesis in GC cells by targeting *c-MYB* [64]. MiR-29a/c was identified as an inhibitory factor of GC angiogenesis. Zhang and coworkers found that transferring exosomes containing overexpressed miR-29a/c remarkably suppressed the growth of vasculature in vivo using a GC tumour implantation mouse model [65].

As the other two vital components of ncRNAs, lncRNAs and circRNAs also exert modulatory effects on GC progression. A high level of lnc-ZFAS1 has been observed in GC tissues, serum specimens and exosomes. Exosomal lnc-ZFAS1 can be transferred into GC cells to increase lnc-ZFAS1 levels and further promote GC cell proliferation and migration [66]. Wang et al. found that silencing exosome-transferred lnc01559 significantly suppressed the proliferation, migration and stemness of GC cells [67]. In addition, Xie et al. found that circSHKBP1 was increased in both GC tissues and serum, and exosomal circSHKBP1 promoted cocultured GC progression by targeting miR-582-3p to increase HUR expression and enhance VEGF signalling [68]. In addition, circSHKBP1 also directly binds to HSP90 and decreases the degradation of HSP90 to accelerate GC progression [68]. Taken together, these findings indicate that exosome-derived miRNAs, lncRNAs and circRNAs can act as functional signals between GC and stroma cells, and enrichment of these signals in GC cells mediated by exosomes contributes to inducing tumorigenesis and disease progression.

### Exosome-derived ncRNAs and GC metastasis

Recurrence and metastasis are the primary obstacles to achieving a good prognosis in GC patients. However, clinically effective biomarkers for identifying metastatic GC are scarce, but recent studies found that exosomal ncRNAs may be novel indicators for identifying metastases with unique advantages. In GC, the most common metastatic location after curative resection is the peritoneum [69, 70], which can be promoted by the mesothelial-to-mesenchymal transition (MMT) [71]. In 2017, Deng et al. first found that GC-derived exosomes induced mesothelial barrier disruption and peritoneal fibrosis by promoting the apoptosis of peritoneal mesothelial cells and enhancing MMT, further facilitating peritoneal metastasis [72]. However, the underlying molecular mechanism has not been revealed. In 2018, Li et al. found that exosomal miR-21 secreted by tumour cells promoted the peritoneal metastasis of GC by regulating the MMT of peritoneal mesothelial cells [73]. Subsequently, a study identified 29 exosomal miRNAs derived from malignant ascites of GC patients by high-throughput sequencing among 8 paired GC patients before and after peritoneal chemotherapy and 3 individuals with non-malignant disease [74]. In addition, GC-derived exosomal miR-106 also induces peritoneal metastasis by regulating the activity of peritoneal mesothelial cells [75].

In addition to peritoneal metastasis, Yang et al. identified remarkedly overexpressed miR-423-5p in the serum exosomes of GC patients, and the analysis of clinicopathological information revealed that exosomal miR-423-5p was related to lymph node metastasis [76]. Mechanistically, exosome-derived miR-423-5p can be internalized into GC cells to promote cell proliferation and migration by targeting suppressors of fused protein (SUFU). Piao et al. found that lncRNA PCGEM1 was specifically expressed in GC exosomes and promoted metastasis by increasing levels of SNAI1, which promotes epithelial-mesenchymal transition (EMT) in GC [77]. In conclusion, exosome-derived ncRNAs regulate metastasis in GC by mediating cellular communication between GC cells and mesothelial cells, enhancing tumour invasive and migratory abilities, MMT, and EMT.

### Immune escape

Activation of the immune system and immune cell development can be suppressed by tumour cell-secreted exosomes to block the immune defence mechanisms of tumour cells. For example, Huber and coworkers found that tumour cells induced the apoptosis of T lymphocytes through the release of Fas ligand-bearing and tumour necrosis factor-related apoptosis-inducing ligand-bearing exosomes both in vitro and in vivo [78]. In addition, Xiang et al. found that differentiation from human myeloid progenitors to dendritic cells can be suppressed by exosomes, which leads to the downregulation of immune system activation, facilitating tumour immune evasion [79].

Recently, several studies have reported that exosomes derived from GC cells participate in immune regulation and influence GC development. For example, M_1_-derived exosomes enhance the T lymphocyte response by downregulating PD-L1 levels [80]. Hinata et al. found that exosomes from Epstein-Barr virus (EBV)-associated gastric carcinoma decreased the fraction of monocyte-derived dendritic cells and then promoted tumour progression [81]. In addition, exosome-mediated communication between tumour cells and immune cells also plays crucial roles in GC development [82]. However, due to limited understanding of the tumour immune microenvironment, the underlying mechanisms need further research.

### Drug resistance

The poor prognosis of GC patients is caused by multiple factors, including resistance to associated drugs. Cisplatin, paclitaxel, 5-fluorouracil and doxorubicin are important chemotherapeutic drugs in the current treatment for GC. A recent study found a high level of miR-21 in exosomes and cell lysates from M_2_ macrophages, and overexpression of miR-21 could be transferred from M_2_ macrophages to GC cells by exosomes to mediate cisplatin resistance [83]. This study was completed in vivo using a subcutaneous transplantation tumour model in nude mice. Mechanistically, exosome-mediated miR-21 transmission increased the levels of miR-21 in GC cells, induced apoptosis and targeted PTEN to activate PI3K/AKT signalling. Notably, tumour cell-secreted exosomal miR-21 promotes MMT, while the exosomal miR-21, which confers cisplatin resistance in GC, originates from tumour-associated macrophages. In addition, exosome-derived miR-155-5p is efficiently taken up by MGC-803 cells, which were sensitive to paclitaxel, subsequently inducing paclitaxel-resistance in an autocrine manner [84]. A high level of circPRRX1 expression was observed in HGC-27 and AGS doxorubicin-resistant GC cell lines, and further research showed that circPRRX1 spread doxorubicin resistance via exosomes [85]. In addition, mechanistic assays found that circPRRX enhanced doxorubicin resistance by sponging miR-3064-5p or regulating expression levels of PTPN14. Similarly, the exosomal lncRNA HOTTIP conferred cisplatin resistance to GC cells by modulating the miR-218/HMGA1 axis [86]. These results reveal the important roles of exosomal ncRNAs originating from both cancer cells and other cells in the TME in modulating the drug resistance of GC cells.

### Exosome-mediated communication between the TME and GC cells

Recently, the roles of the tumour-associated microenvironment, primarily consisting of bone marrow-derived cells [87, 88], tumour-associated mast cells [89, 90], cancer-associated fibroblasts (CAFs) [91, 92], cancer-associated macrophages (CAMs) [93], tumour-infiltrating neutrophils [94], miRNAs [95], exosomes [96] and extracellular matrix [97, 98], have attracted growing attention among researchers, greatly deepening the understanding of cancer development. Among these components, exosomes not only directly regulate GC cell biology but also act as mediators for communication between the TME and cancer cells. For example, given that the hypoxic TME is a common characteristic of nearly all solid tumours [99], many oxygen modulation-related genes are activated to respond to hypoxia and maintain the normal physiological activities of cancer cells [100]. A hypoxic TME in GC leads to the upregulation of miR-301a-3p and promotes its secretion through exosomes [101]. Then, exosomal miR-301a-3p is ingested by GC cells, and increased miR-301a-3p inhibits HIF-1a degradation by targeting PHD3 [101]. Finally, this positive feedback loop promotes GC progression and metastasis. The detailed interactions between other TME contents and GC cells are further discussed below.

### Exosomal ncRNA-mediated crosstalk between CAFs and GC cells

**Fig. 4:**
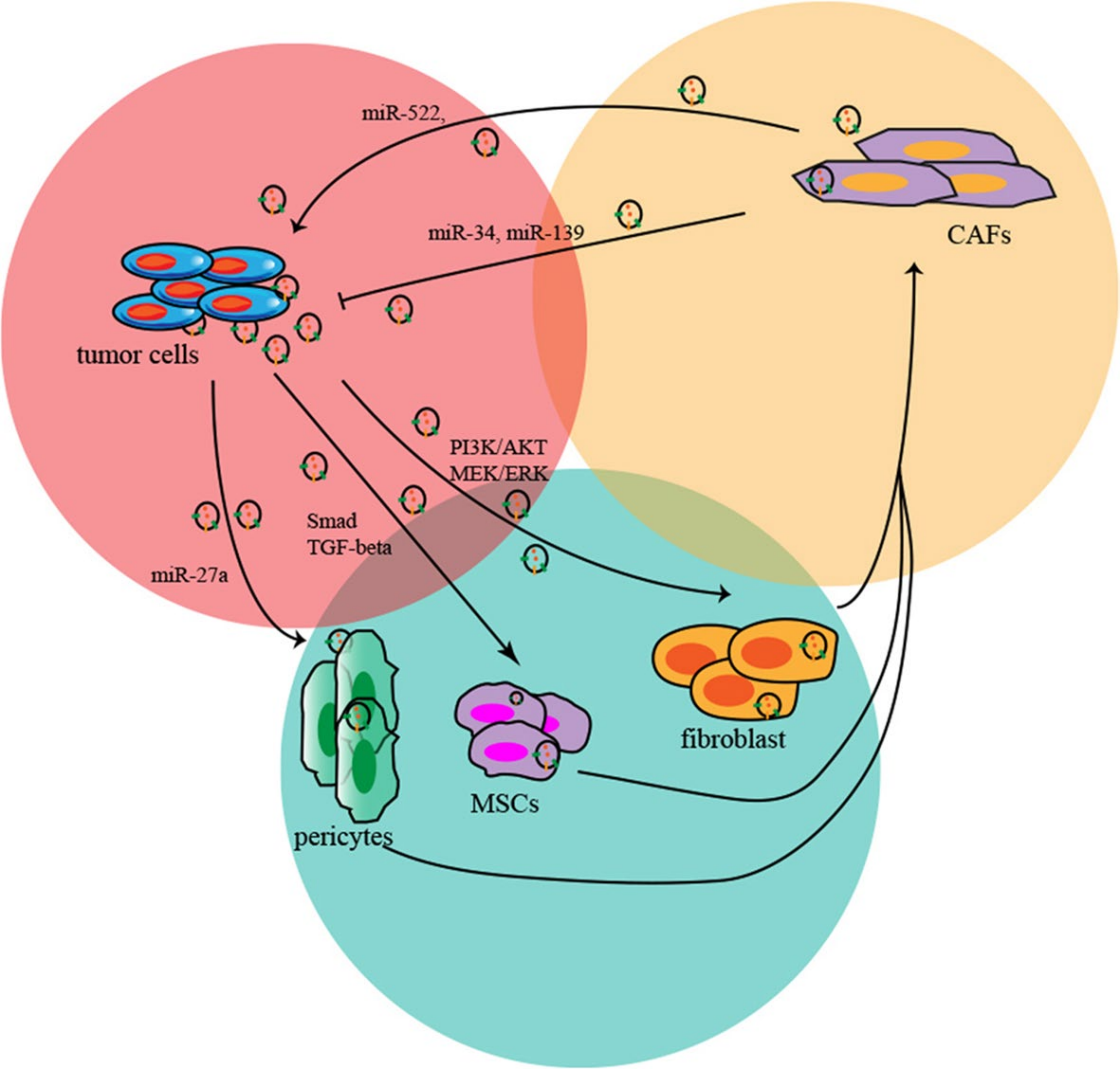
Exosomal ncRNA-mediated crosstalk between CAFs and GC cells. GC cell-derived exosomes promote the differentiation of CAFs from pericytes, MSCs and fibroblasts. CAFs modulate the progression of GC through exosome-derived miR-34, miR-139 and miR-522

Recent studies found that there are various stromal cells in the primary tumour bed of GC, in addition to GC cells. Among them, CAFs are a major component characterized by heterogeneous spindle-shaped groups, which are primary derived from local fibroblasts, mesenchymal stem cells, endothelial cells, epithelial cells, adipocytes and pericytes [102]. The exosomal molecule-mediated reciprocal feedback loop is an important communication mode between CAFs and cancer cells (Fig. 4). Tumour-secreted exosomal molecules induce fibroblast activation, and activated fibroblasts induce the growth and metastasis of cancer. For example, GC cells induce the differentiation of hucMSCs to CAFs by exosome-mediated inhibition of Smad-2 phosphorylation and TGF-β transfer [103]. GC exosomal miR-27a (exomiR-27a) induces local fibroblasts to transform into CAFs [104]. GC cells can also remarkably induce the reprogramming of pericytes to CAFs by exosome-mediated MEK/ERK and PI3K/AKT signalling pathway activation [105]. In addition, CAFs also regulate GC progression by exosome-mediated molecular communication. Recent studies have revealed that ferroptosis is associated with the efficacy of chemotherapy in cancer [106, 107]. Exosomal miR-522 derived from CAFs decreased the expression levels of lipid ROS by targeting arachidonate lipoxygenase 15 (ALOX15) in GC, and downregulated lipid ROS suppressed ferroptosis and promoted acquired chemoresistance [108]. Miki et al. found that CD9-positive exosomes derived from CAFs enhanced the migration of scirrhous-type GC cells [109].

However, antitumor effects were also observed in CAF-secreted exosomes. For example, exosomal miRNA-34 and miR-139 derived from CAFs suppressed the malignant behaviours and metastasis of GC cells in vitro and in vivo [110]. Coculture of GC cells and CAFs with overexpressed miRNA-34 led to suppression of the invasion and migration of GC cells [110]. Exosomal miR-139 suppressed the progression and metastasis of GC by downregulating levels of MMP11 [111]. Thus, CAFs play an important role in the oncogenesis and development of GC, and anti-CAF agents targeting related exosomal molecules may represent a potential therapeutic strategy.

### Exosomal ncRNA-mediated interactions between macrophages and GC cells

**Fig. 5:**
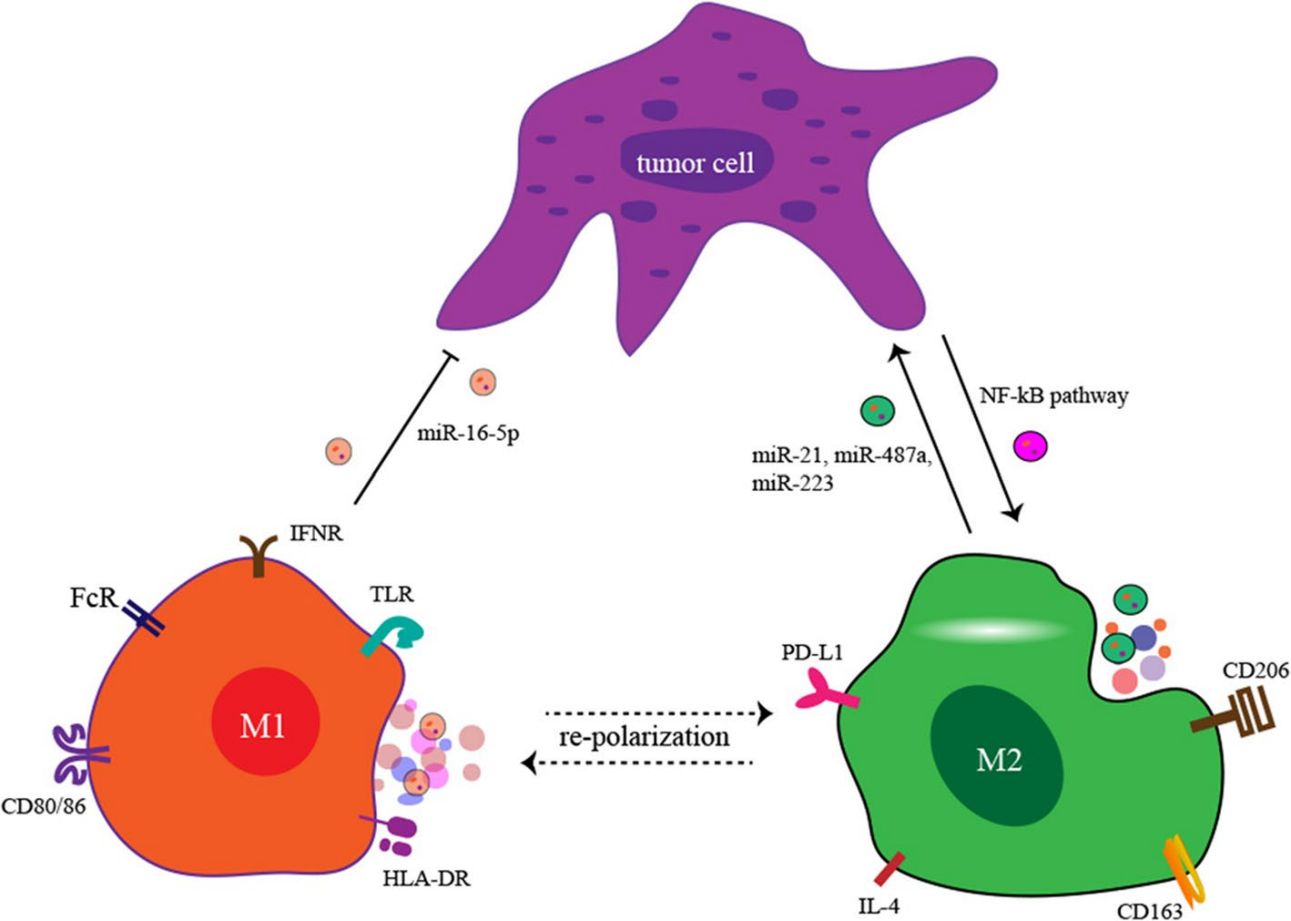
Exosomal ncRNA-mediated interactions between macrophages and GC cells. GC cell-derived exosomes promote the M_2_ polarization of macrophages through the NF-κB signalling pathway. M_2_ cells promote the progression of GC through exosome-derived miR-21, miR-487a and miR-223. M_1_ macrophages suppress the proliferation of GC cells through exosome-derived miR-16-5p

Macrophages are the primary phagocytes in vivo and exert their effects by engulfing cellular debris, intracellular parasites, bacteria, apoptotic cells, cancer cells, and ageing and abnormal cells [112]. In the TME, macrophages are the most numerous stromal leukocytes with two differential subtypes, M_1_ and M_2_. M_1_ cells have a pro-inflammatory function with antitumor effects, and the M_2_ subtype of macrophages acts as anti-inflammatory cells that are associated with tumour growth and metastasis [113]. Previous studies found that exosomal molecules from GC cells can regulate the polarization of macrophages, and polarized macrophages influence cancer progression and metastasis (Fig. 5). Wu et al. found that exosomes derived from GC cells could stimulate macrophages polarized to the M_2_ subtype by activating the NF-κB pathway [114]. In addition, another study found that SGC-7901 and BGC-823 cell-secreted exosomes induced monocytes to differentiate into PD-1-positive tumour-associated macrophages with M_2_ cell characteristics. These PD-1-positive tumour-associated macrophages produced numerous IL-10 molecules to impair the function of CD8^+^ T cells and further promote GC progression. In GC cells, exosomal transfer of M_2_ polarized macrophages secreting miR-21 and miR-233 conferred chemotherapy resistance to cisplatin and doxorubicin, respectively [83, 115]. Exosomal miR-21 can be directly transferred from M_2_ cells to GC cells and then activate the PI3K/Akt pathway by decreasing PTEN. In addition, M_2_ cells can promote GC progression and metastasis by delivering exosomes containing miR-487a [116], apolipoprotein E protein [117] and miR-223 [118]. In addition, the effects of exosomal miRNAs from M_1_ polarized macrophages on GC cells have also been investigated. Li et al. reported that M_1_ macrophage-secreted exosomes carrying miR-16-5p reduced the expression of PD-L1 in GC cells to induce a T cell-dependent immune response, exerting anticancer effects [80]. Therefore, redirecting the polarization of macrophages in GC may represent a novel therapeutic strategy.

### Exosomal ncRNA-mediated interactions between neutrophils and GC cells

Neutrophils are vital regulators in cancer initiation and progression [119, 120]. Previous studies have shown that the presence of neutrophils in GC is associated with lymph node metastasis and predicts a poor prognosis [121]. Zhang and colleagues found that conditioned medium from GC cells induced autophagy and N2 polarization of neutrophils, which promoted cancer progression. Further study on the mechanism revealed that GC cell-derived exosomes induced neutrophil activation through the NF-κB pathway [82]. Subsequently, Shi et al. found that GC cell-derived exosomes significantly increased the expression levels of PD-L1 in neutrophils to suppress the T lymphocyte-based immune response [122].

### Clinical applications

Because exosome-derived ncRNAs play a vital role in GC, studies on their functions and mechanisms indicate promising clinical applications. Based on current research, there are four primary applications: diagnostic biomarkers, prognostic biomarkers, therapeutic targets and reversal of chemoresistance (Table 1).

**Table 1:** Exosomal ncRNA-associated clinical applications

Clinical applications	Exosomal ncRNA	Origin of exosomes	Tendency	Downstream target	Role
Diagnostic biomarker	LncRNA-GC1	Plasma	Up	NA	Screen early-satge GC and monitor disease progression
	MiR-106a-5p	Serum	Up	NA	Screen GC
	MiR-19b-3p	Serum	Up	NA	Screen GC
	LncUEGC1	Plasma	Up	NA	Screen early-satge GC
	MiR-15b-3p	Serum	Up	DYNLT1	Screen GC
	Lnc-GNAQ-6	Serum	Down	NA	Screen GC
	Linc-00152	Plasma	Down	NA	Screen early-satge GC
	LncHOTTIP	Serum	Up	NA	Screen GC
Prognostic biomarker	MiR-15b-3p	Serum	Up	DYNLT1	Predict OS
	MiR 23b	Plasma	Down	NA	Predict recurrence, OS and DFS
	MiR-21	Peritoneal lavage fluid	Up	NA	Predict peritoneal metastasis
	MiR-1225-5p	Peritoneal lavage fluid	Up	NA	Predict peritoneal metastasis
	MiR-29 s	Peritoneal lavage fluid	Down	NA	Predict peritoneal recurrence
	MiR-423-5p	Serum	Up	SUFU	Predict lymph node metastasis
	MiR-10b-5p	Plasma	Up	NA	Predict lymph node metastasis
	MiR-143-5p	Plasma	Up	NA	Predict liver metastasis
	MiR-101-3p	Plasma	Up	NA	Predict ovarian metastasis
	LncHOTTIP	Serum	Up	NA	Predict OS
Therapeutic target	MiR-21	Macrophage culture supernatants	Up	PDCD4	Promote the proliferation, invasion and migration of GC cells, and induce cell apoptosis
Reverse chemo-resistance	MiR-214	GC cells culture supernatants	Up	NA	Induce cisplatin resistance
	MiR-374a-5p	GC cells culture supernatants	Up	Neurod1	Induce oxaliplatin resistance

*Abbreviations*: *GC* gastric cancer, *NA* not avaliable, *DYNLT1* dynein light chain tctex-type 1, *SUFU* suppressor of fused protein, *PDCD4* programmed cell death 4, *OS* overall survival, *DFS* disease-free survival

### Diagnostic biomarker

Current clinical diagnostic biomarkers for GC, such as CEA, CA19-9, CA724 and CA242, are not sensitive or specific enough to screen patients with GC [123, 124]. Therefore, it is urgent to develop simple, novel and effective biomarkers for the detection of GC. Recently, several exosome-derived ncRNAs have attracted increasing attention. Due to their location in various body fluids, exosome-related examination is more convenient and is non-invasive.

Hoshino et al. analysed the proteomic profile of exosomes in 426 human specimens from plasma, other bodily fluids and tissue explants, and they found that comparison of plasma exosomes can distinguish cancer from normal tissues with specificities and sensitivities of 95% and 90%, respectively [125]. A multiphase study with 826 enrolled patients found that almost all circulating lncRNA-GC1 was packaged into exosomes [126]. Moreover, circulating lncRNA-GC1 can survive RNase, room temperature and repeated freezing and thawing. The analytic results showed that using lncRNA-GC1 to screen for GC had a better predictive value than conventional biomarkers, including CEA, CA-199 and CA-724 [126]. In addition, microarray profiles identified that miR-106a-5p and miR-19b-3p were remarkably overexpressed in the serum exosomes of patients with GC. Notably, integrating the two miRNAs could identify GC patients among healthy volunteers with a 0.814 area under the curve (AUC) value, which was higher than that obtained using CEA or AFP [127]. Moreover, lncUEGC1 could be used for screening early-stage GC [128]. Exosome-derived miR-15b-3p, lnc-GNAQ-6, linc-00152 and lncHOTTIP were also identified to have promising diagnostic value in GC patients [62, 129–131]. Of note, Ge and colleagues first identified exosome-derived P-element-induced wimpy testis (PIWI)-interacting RNAs (piRNAs), which were more abundant than miRNAs in cells and could be used as non-invasive diagnostic biomarkers with high sensitivity and specificity [132]. Taken together, exosome-derived ncRNAs can serve as sensitive and specific non-invasive biomarkers for GC screening.

### Prognostic biomarker

Recurrence and metastasis are related to the poor prognosis of patients with GC. In miRNA microarray of exosomal miRNAs from 3 healthy volunteers and 6 GC patients with or without recurrence, Kumata and coworkers identified deregulated exosomal miRNAs, and miR-23b exhibited the most significant change [133]. Subsequently, they validated the prognostic value of miR-23b in 232 random GC patients and 20 healthy volunteers. The results showed that exosomal miR-23b was significantly related to tumour size, depth of invasion, liver metastasis and TNM stage. In addition, the results revealed that exosome-transmitted miR-23b represented a predictive biomarker for overall survival and disease-free survival. Peritoneal metastasis is the most common metastatic location of GC, and patients with peritoneal metastasis usually have shorter overall survival. Several exosomal miRNAs were detected at high levels in peritoneal lavage fluids, malignant ascites and culture medium of GC cells. Among them, miR-21, miR-1225-5p and miR-29 s were identified to be related to serosal invasion, providing predictive factors for the early diagnosis of peritoneal metastasis in GC [134, 135]. Ohzawa and coworkers detected a relatively low level of exosomal miR-29 s in peritoneal lavage fluid or ascites of GC patients with peritoneal metastases compared to patients without peritoneal metastases [135]. Further research showed that GC patients with high levels of exosomal miR-29 s tended to develop peritoneal recurrence after curative gastrectomy, and levels of exosomal miR-29 s were associated with overall survival [135]. Yang et al. found that exosomal miR-423-5p was related to lymph node metastasis, and high levels of miR-423-5p indicated poor prognosis in GC patients [76]. In addition, through small RNA sequencing and conformation by clinical plasma samples, exosomal miR-10b-5p, miR-143-5p and miR-101-3p were identified as indicators of GC with lymph node metastasis, GC with liver metastasis and GC with ovarian metastasis, respectively [136].

### Therapeutic target

In 2011, Zhang et al. first proposed the therapeutic potential of exosomes in GC. They demonstrated that malignant ascites-derived exosomes induced by heat treatment promoted dendritic cell maturation and the T cell-associated immune response [137]. Therefore, they predicted that these exosomes could be used as an effective tumour vaccine in GC. In addition, current studies have revealed that exosomes and exosomal ncRNAs, whether secreted by tumour cells or other cells in the TME, primarily exert oncogenic effects in GC. As such, blocking the release of these exosomes may suppress the development of GC. In 2017, Guan and colleagues confirmed that proton pump inhibitors suppressed the release of exosomal miRNAs in GC to exert anticancer effects [138]. Another study found that a miR-21 inhibitor loaded in exosomes significantly suppressed the proliferation, invasion and migration of GC cells by inducing apoptosis and suppressing cell migration [139]. These results reveal a new antitumor strategy using ncRNAs containing exosomes to control or block GC growth, metastasis and drug resistance.

### Reversing chemo-resistance by delivering ncRNAs

Exosomes can also be used to deliver molecules or drugs for treating GC. Compared with viral vectors, exosomes are natural nanocarriers secreted by endogenous cells with low immunogenicity and cytotoxicity [140]. Besides, exosomes can protect the containing ncRNAs from RNase [141]. Furthermore, exosome-mediated molecular transfers decreased the accumulation of loaded ncRNA in nontarget tissues, therefore reducing off-target toxicity [142]. Recently, Sun and coworkers found that chemoresistant GC cells promoted the chemoresistance of chemosensitive GC cells via exosome-mediated substance exchange [143].Inhibiting this substance exchange mediated by exosomes or associated molecules may exert an antitumor role in GC. In 2018, Wand and colleagues found that transferring exosomes carrying anti-miR-214 into GC cells reversed cisplatin resistance [144]. Subsequent studies revealed that transferring exosomes carrying miR-374a-5p and c-MET siRNA in GC cells also reversed chemoresistance to oxaliplatin and cisplatin, respectively [145, 146]. These findings propose a novel target for drug resistance therapy in GC.

### Conclusions and future perspectives

Exosome-derived ncRNAs are key regulators in the complex communication between the TME and GC cells and exert crucial effects on the direction of stromal cell differentiation, such as macrophages polarized to M2 and neutrophils differentiated to N2, and tumour development, indicating that they are important targets for blocking or eradicating GC. In addition, nearly all types of cells can release and internalize exosomes. Therefore, developing drugs targeting exosomes or exosomal ncRNAs may represent a novel method to treat GC. Additionally, loading tumour suppressors or molecules targeting oncogenic ncRNAs into exosomes and transferring these exosomes to GC are potential strategies for treatment, and the application of exosomes carrying anti-miR-214 and miR-21 inhibitors has achieved success.

The good stability of exosomal ncRNAs in body fluids, due to the membrane structure of exosomes, allows for novel non-invasive detection to screen GC patients and evaluate prognosis at an earlier stage. Current studies referring to liquid biopsies include the detection of circulating DNA, circulating tumour cells, exosomes and miRNAs. Of these, there are several unique advantages to examining exosomes. For example, the efficient transfer of particular exosomal DNA/RNA/protein profiles to tumour cells allows for early detection of GC by assessing specific biomarkers. However, the most suitable component needs further identification, and the specific methods for precise application in vivo also need further exploration to achieve higher precision and decrease side effects to nontarget organs.

Although remarkable advances have been made in the understanding of exosomes and their cargo, some challenges remain. For example, a standard method for isolating exosomes or ectosomes from various biofluids is lacking. Different isolation methods may result in different subpopulations of extracellular vesicles with different miRNAs, proteins, diameters and functions [147–149]. In addition, there is a lack of data concerning the molecular constituents and quantity of exosomes in nondisease samples. Furthermore, it is unclear how clinical features, such as age, race and sex, affect the contents of exosomes, and it is imperative to characterize these associations to further research the role of exosomes in human disease occurrence and progression. It is not clear how to control contamination of nonvesicular macromolecules in exosome isolation, such as lipoproteins [150]. In addition, nearly all studies have focused on cellular experiments, and the safety and efficacy of exosome-associated applications in vivo need further research. In summary, continued in-depth investigations to understand the effects and mechanisms of exosomal ncRNAs are required to develop exosome-associated strategies for GC diagnosis and treatment.

## Data Availability

Not applicable.

## Abbreviations

TME: Tumor microenvironment
ncRNAs: Non-coding RNAs
GC: Gastric cancer
ILVs: Intraluminal vesicles
EVs: Extracellular vesicles
MVBs: Multivesicular bodies
CSF: Cerebral spinal fluid
hnRNPA2B1: Heterogeneous nuclear ribonucleoprotein A2B1
mRNA: Messeger RNA
miRNA: MicroRNA
lncRNA: Long non-coding RNA
circRNA: Circular RNA
MVP: Major vault protein
ESCRT: Endosomal sorting complex required for transport
USP8: Ubiquitin-specific peptidase 8
AICD: APP intracellular domain
SNARE: Sensitive factor attachment protein receptor
TfR: Transferrin receptors
MHC: Major histocompatibility complex
ALIX: ALG2-interacting protein X
HSP70: Heat shock protein 70
TSG101: Tumor susceptibility gene 101 protein
GC-MSCs: GC tissue-derived mesenchymal stem cells
hucMSCs: Human umbilical cord-derived MSCs
SUFU: Suppressors of fused protein
MTT: Mesothelial-to-mesenchymal transition
EMT: Epithelial-mesenchymal transition
CAMs: Cancer-associated macrophages
exomiR-27a: Exosomal miR-27a
ALOX15: Arachidonate lipoxygenase 15
AUC: Area under curve
PIWI: P-element-induced wimpy testis
piRNAs: PIWI-interacting RNAs
